# Intraocular lens iris fixation. Clinical and macular OCT outcomes

**DOI:** 10.1186/1756-0500-5-560

**Published:** 2012-10-10

**Authors:** Leonardo Garcia-Rojas, Juan Manuel Paulin-Huerta, Eduardo Chavez-Mondragon, Arturo Ramirez-Miranda

**Affiliations:** 1 Anterior Segment Department, Instituto De Oftalmología Fundación Conde De Valenciana, Mexico City, Mexico; 2 Cornea and Refractive Surgery Department, Instituto De Oftalmología Fundación Conde De Valenciana, Chimalpopoca 14 Colonia Obrera, Mexico City 06800, Mexico

## Abstract

**Background:**

To assess the efficacy, clinical outcomes, visual acuity (VA), incidence of adverse effects, and complications of peripheral iris fixation of 3-piece acrylic IOLs in eyes lacking capsular support. Thirteen patients who underwent implantation and peripheral iris fixation of a 3-piece foldable acrylic PC IOL for aphakia in the absence of capsular support were followed after surgery. Clinical outcomes and macular SD-OCT (Cirrus OCT; Carl Zeiss Meditec, Germany) were analyzed.

**Findings:**

The final CDVA was 20/40 or better in 8 eyes (62%), 20/60 or better in 12 eyes (92%), and one case of 20/80 due to corneal astigmatism and mild persistent edema. No intraoperative complications were reported. There were seven cases of medically controlled ocular hypertension after surgery due to the presence of viscoelastic in the AC. There were no cases of cystoid macular edema, chronic iridocyclitis, IOL subluxation, pigment dispersion, or glaucoma. Macular edema did not develop in any case by means of SD-OCT.

**Conclusions:**

We think that this technique for iris suture fixation provides safe and effective results. Patients had substantial improvements in UDVA and CDVA. This surgical strategy may be individualized however; age, cornea status, angle structures, iris anatomy, and glaucoma are important considerations in selecting candidates for an appropriate IOL fixation method.

## Findings

### Introduction

Cataract surgery is the most common intraocular surgery; about 10 million cataract surgeries are performed worldwide each year [1]. Despite the low rates of surgical complications, aphakia and malpositioned intraocular lenses (IOLs) in the absence of capsular support represent a clinical problem and a surgical challenge. Anterior chamber (AC) IOLs, posterior chamber (PC) trans-scleral sutured IOLs, and PC iris-fixated IOLs are commonly used surgical approaches to treat aphakia and malpositioned IOLs. A review by the American Academy of Ophthalmology concluded that there was insufficient evidence to demonstrate superiority of one type or fixation site over another [2]. Although modern AC IOLs designs have significantly improved, concerns about corneal decompensation, trabecular meshwork damage, and chronic inflammation still exist [3,4]. Scleral fixation PC IOLs avoids some of these problems. However, disadvantages such as IOL tilting, vitreous entrapment, retinal detachment, intraocular hemorrhage, and a technically challenging surgery, appears as a questionable alternative [5-10]. Iris suture fixation of an IOL represents an alternative for those cases in which anterior segment anatomy is preserved. Iris-fixated PC IOLs approximates the anatomical position of a capsule supported IOL, they are located far from the corneal endothelium and the trabecular meshwork; however, concerns still about iris chafing, pigment dispersion, chronic inflammation, and peripheral anterior synechiae [11-14].

The purpose of the current study is to assess the efficacy, clinical outcomes, visual acuity (VA), incidence of adverse effects, and complications of peripheral iris fixation of 3-piece acrylic IOLs in eyes lacking capsular support.

### Materials and methods

After obtaining local Institutional Ethics Committee approval and informed consent from the participants, thirteen patients who underwent implantation and peripheral iris fixation of a 3-piece foldable acrylic PC IOL were followed after surgery. Preoperative diagnosis, intraoperative events, postoperative visual acuity, clinical outcomes and macular Spectral Domain Optical Coherence Tomography (SD-OCT; Cirrus OCT, Carl Zeiss Meditec, Germany) were analyzed. Follow up examinations were performed at a monthly basis.

**Figure 1:**
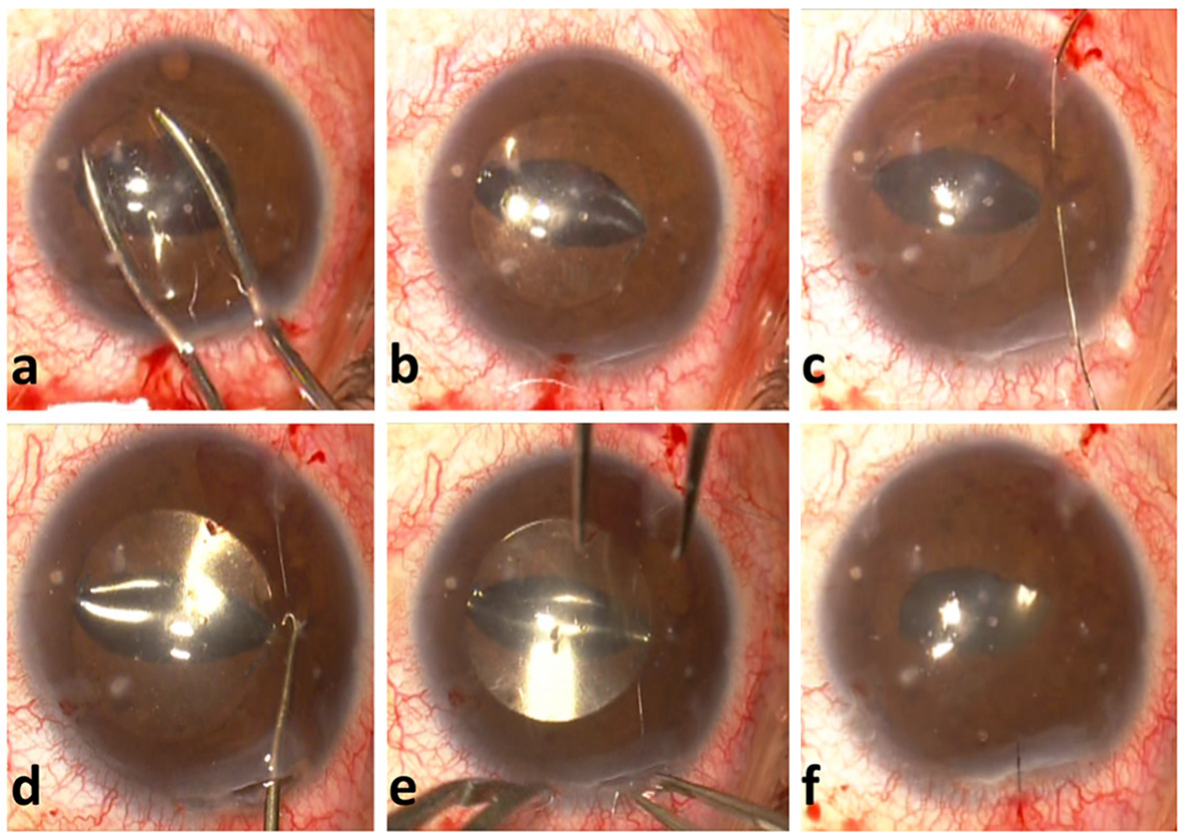
**a. Folded 3-piece acrylic PC IOL.b**. Optic captured in the AC above iris plane and both haptics behind it. **c to e**. Modified McCannel fixation using Siepser slipknot with a 9–0 polypropylene suture. **f**. Once both haptics were sutured, the optic was prolapsed into the PC.

Under retrobulbar anesthesia, a 4.2 mm clear corneal incision was made superiorly, acetylcholine (Iloc, Sophia Laboratories, Guadalajara Mexico) was placed in the AC followed by a combination of 4% sodium chondroitin sulfate and 1.65% sodium hyaluronate Ophthalmic Viscosurgical Device (OVD) (DiscoVisc, Alcon Laboratories, Ft. Worth, Texas) avoiding posterior iris displacement and pupillary expansion. Anterior vitrectomy was performed as needed to clear the retropupillary space before IOL implantation. A 3-piece acrylic PC IOL (Acrysof® MA60AC Alcon Laboratories, Ft. Worth, Texas) was folded and placed in the AC, with the optic captured in the AC above iris plane and both haptics behind it. Modified McCannel [15] sutures using Siepser [16] slipknot were used to fixate each haptic to the peripheral iris with a 9–0 polypropylene suture on a long gently curved needle (Visionary Medical Supplies, INC, Madison ,Wisconsin) retrieved through corneal paracentesis. Once both haptics were sutured, the optic was prolapsed into the PC, OVD was removed by washing the AC with 15 ml of Balanced Salt Solution, and wound was closed with a 10–0 nylon suture (Figure 1). All patients were treated with topical medication (moxifloxacin hydrochloride for 10 days, prednisolone acetate tapered for 2 weeks and sodium nepafenac for 6 weeks); ocular hypotensors were prescribed as needed.

Statistical analysis was performed using Excel® (Microsoft, Inc.) and SPSS v.17. A 2-tailed distribution *t* test was used for evaluating differences in mean preoperative and postoperative VA and foveal thickness. All Snellen VAs were converted to logarithm of the minimum angle of resolution values for statistical analysis.

### Results

**Figure 2:**
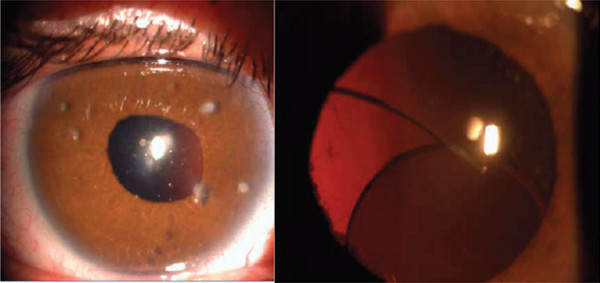
**Left: Aphakia secondary to trauma.** Right: Posterior Chamber IOL subluxation.

Thirteen eyes from thirteen patients underwent successful implantation of an iris fixated PC IOL. Mean age at time of surgery was 62.53 years (range 28–86), four were female and nine were male. Surgery was indicated due to aphakia without capsular support in 9 cases and due to IOL dislocation in 4 cases (Figure 2).

**Figure 3:**
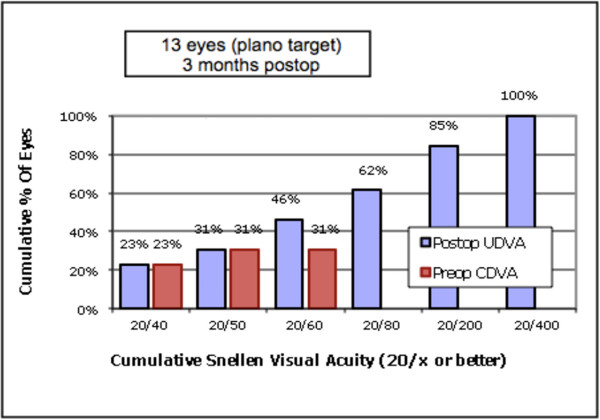
**Cumulative Snellen Visual Acuity.** Preoperative Corrected Distance Visual Acuity (CDVA) and Postoperative Uncorrected Distance Visual Acuity (UDVA), showing the efficacy and visual improvement.

All patients were followed at least for 3 months after surgery. Nine patients improved their uncorrected distance visual acuity (UDVA) and retained or improved their preoperative corrected distance visual acuity (CDVA). Mean preoperative UDVA and CDVA significantly improved postoperatively (LogMAR 1.91 and 1.00 vs. 0.66 and 0.30, respectively; *P* < 0.01) (Figure 3).

Combined anterior vitrectomy and IOL fixation at the same surgical event was performed in four cases. Intraoperatively, there were no instances of bleeding, iridodialysis, IOL dislocation or any other surgical complication.

**Figure 4:**
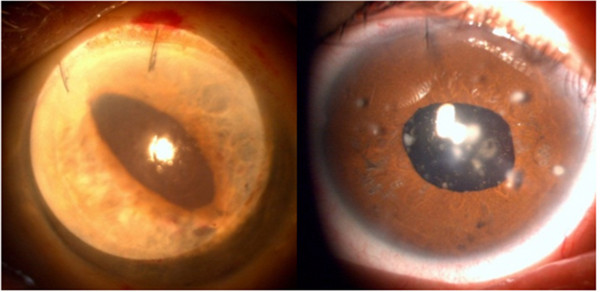
**Different grades of pupil ovalization.** Left: Oval pupil due to haptic sutures. Right: Round pupil.

The final CDVA was 20/40 or better in 8 eyes (62%), 20/60 or better in 12 eyes (92%), and one case of 20/80 due to corneal astigmatism and mild persistent edema. In all 13 eyes, the pupil was reactive, 5 were round and 8 had different grades of ovalization (Figure 4).

There were seven cases of medically controlled ocular hypertension after surgery due to the presence of viscoelastic in the AC. There were no cases of cystoid macular edema, chronic iridocyclitis, IOL subluxation, pigment dispersion, or glaucoma. The mean follow-up was 4.86 months, with a minimum of 3 months.

Macular edema did not develop in any case by means of SD-OCT. Foveal thickness showed a non-significant increase; preoperative and postoperative foveal thickness values were 211 vs. 214 μm (*P* >0.05).

### Discussion

IOL selection and implantation method for the correction of aphakia, in eyes without capsular support continues to be a matter of controversy. Most published studies have been uncontrolled, with an inherent selection bias and varying patient populations. Only one randomized trial comparing the 3 IOL fixation strategies has been published [4]. This makes difficult to conclude how to correct aphakia in a safe and efficient way. Patient age, cornea status, angle structures, iris anatomy, and coexisting glaucoma are important considerations in selecting candidates for this surgeries and an appropriate fixation method. There is maybe not only one option for all cases, therefore each case must be studied and individualized.

Anterior chamber IOL implantation might be the simplest procedure for surgical aphakia correction. However, with the proximity of lens haptics to the cornea, there is concern for corneal decompensation, glaucoma, and chronic inflammation [3,4]. Correct sizing for AC angle width is critical to prevent IOL rotation and/or corneal contact or iris entrapment with the subsequent chronic inflammation. Recent imaging studies with high-speed optical coherence tomography have found that this method is relatively inaccurate and has a lack of correlation, thus creating uncertainty with AC IOL implantation [17]. Furthermore, a relatively large incision of at least 6 mm is required for most of the currently available AC IOLs, perhaps, in the near future a foldable hydrophobic acrylic AC IOL with an adequate distance from the cornea will be available in an aphakic power range [18].

Scleral sulcus-fixated PC IOL implantation, although technically more demanding has the advantages of avoiding some of the corneal, angle, and sizing issues present in the AC IOL approach. However, unavoidable risks with transscleral suturing include: intraoperative hemorrhage, externalized suture, late suture breakage, suture tract–related endophthalmitis, IOL tilting, optic capture, and peripheral anterior synechiae formation [5-10]. Furthermore, ultrasound biomicroscopy studies have found considerable variability in haptic position, with the majority of haptics found in locations other than the intended ciliary sulcus, and many occasions with vitreous incarceration [19,20].

Iris-sutured PC IOL implantation retains the potential benefits of a PC IOL and avoids the risks associated with transscleral external sutures. In cases of megalocornea, with an excessively large AC and ciliary sulcus, AC IOL, endocapsular PC IOL, or sulcus-sutured PC IOL fixation would increase the risk of decentration, thus making iris-fixated IOLs the ideal choice [21].

A report from the American Academy of Ophthalmology reviewed the literature on open-loop AC IOL, iris-sutured PC IOL, and scleral-sutured PC IOL implantation in the absence of capsule support and concluded that there was insufficient evidence to demonstrate the superiority of one lens type or fixation site over the other [2]. Many of these studies were in the setting of concurrent penetrating keratoplasty (PK), thus resulting in a major confounding factor in evaluating visual outcome and complications. Indeed, of 8 articles on iris-sutured PC IOLs reviewed by the American Academy of Ophthalmology, 5 were in conjunction with PK. Postoperative complications, including cystoid macular edema (CME), glaucoma progression, and corneal decompensation, are all greater when IOL implantation is combined with PK as opposed to without it [2], thus making it difficult to evaluate the efficacy and safety of the IOL fixation technique independently.

Just one randomized trial comparing the 3 IOL fixation strategies has been published. This was conducted with concomitant PK in 176 patients lacking adequate capsule support [22]. Although visual outcomes were similar for the 3 groups, it was found that iris-sutured PC IOLs were associated with significantly less cystoid macular edema (20%) versus AC IOLs (38%) or scleral-sutured PC IOLs (41%) (*P* _ 0.02) [22]. Scleral-sutured IOLs were found to have the highest overall number of complications [22].

Condon et al., reported the largest series with a 46 patients study that underwent a successful implantation and iris fixation of a foldable acrylic PC IOL with a mean follow-up of 24.1±12.4 months [23]. Forty-four of 46 patients (95.7%) either retained or improved their CDVA; two patients lost CDVA due to bullous keratopathy and epiretinal membrane formation, respectively [23]. Thirty-two of 46 (69.6%) eyes achieved a CDVA of 20/40 or better, with 41 of 46 (89.1%) reaching 20/80 or better after surgery [23]. Postoperative complications included two IOL dislocations, three cases of prolonged mild uveitis that resolved with topical steroids, three patients developed pigment dispersion, one of whom developed elevated intraocular pressure controlled with topical antiglaucoma medications, and no CME cases [23]. Navia-Array, reported excellent visual outcomes in 30 cases utilizing a limbal approach with a specially designed rigid 4-hole optic PC IOL, a large 7 mm incision, and suture fixation of the IOL optic to the midperiphery iris, in which 63% of the patients achieved a VA of 20/40 or better with no serious anterior segment complications [13]. Four patients had mild pigment dispersion, and 1 had persistent CME. No patients developed corneal edema, IOL subluxation, or endophthalmitis with a mean follow-up of 40 months. Hoh et al., reported an increase in VA after iris/suture fixation of a 7 mm 2-hole optic IOL in 27 of 30 eyes, with no major complications [12]. Zeh and Price, also found satisfactory results in 28 eyes that had iris fixation of a PC IOL through a limbal incision, with 82.1% achieving VA of 20/80 or better and 57.1% achieving 20/40 or better [24]. From these studies, concerns relating to pigment dispersion, glaucoma progression, chronic iritis, CME, or corneal decompensation, appear to be minimal and no greater than with alternative fixation strategies. Although some degree of immediate iris pigment dispersion occurs perioperatively with iris fixation of a PC IOL, progressive dispersion glaucoma, has not been identified as a common late complication.

In our study, with both, clinical and SD-OCT mean follow-up of 4.8 months (3 to 6 months) of 13 eyes, we found that this small-incision technique of foldable acrylic IOL iris fixation could be a reproducible, safe, and effective technique. Patients had substantial improvements in UDVA and CDVA. Sixty-two percent of eyes achieved CDVA of 20/40 or better and 92% of eyes achieved 20/60 or better.

The previously used surgical technique, combining the use of a small-incision acrylic foldable IOL inserted in a moustache-fold fashion and fixation with a polypropylene suture on long curved needles with the McCannel retrievable suture concept [15], is a simpler and less technically challenging limbal approach that has been popularized [25-27]. Adding Siepser [16] slipknot has been a further modification by Condon et al. series [23], and our case series promote more secure fixation and reduce haptic slippage [12,13,22]. Considering pars plana or limbal vitrectomy to free the AC and retropupillary space of vitreous when present is mandatory.

This combined surgical technique has significant advantages over others in the following senses: small incision, secure optic IOL positioning during suture passage, easily needle pass in stable midperipheral iris, and avoidance of suture contact with rigid polymethyl methacrylate that could result in late suture breakage. Furthermore, there is a very low risk of bleeding, there is no or minimal conjunctival manipulation, fixing angulated haptics to the peripheral iris may result in less anterior displacement of the peripheral iris, which allows the IOL to have a slight posterior vaulting, and surgery is achievable under retrobulbar anesthesia.

Our study is limited by the short case series and the relatively short follow-up. Although some cases were followed up to 36 months clinically, macular SD-OCT was performed up to six months after surgery.

We cannot exclude selection bias: patients who were more likely to benefit from iris fixation may be represented more. It is important to note that no patients had an unstable iris, significant iris abnormalities, or large iris defects that may be a contraindication to iris suture fixation.

Further randomized clinical trials comparing data of individualized procedures of open-loop AC IOL, iris-sutured PC IOL, and scleral-sutured PC IOL implantation in the absence of capsule support are required to determine long-term efficacy and safety.

In summary, our short-term results revealed excellent visual outcomes and safety with iris-sutured foldable acrylic lenses. These results are either comparable to or better than those in the current literature on IOL fixation strategies for eyes lacking capsule support. Compared with an AC IOL or scleral fixation of a PC IOL to correct aphakia, we think that this technique for iris suture fixation provides safe and effective results. This surgical strategy may be individualized however; age, cornea status, angle structures, iris anatomy, and glaucoma are important considerations in selecting candidates for an appropriate IOL fixation method.

## Competing interests

The authors report no conflicts of interest. The authors alone are responsible for the content and writing of the paper. No financial support was received.

## Authors’ contributions

LGR contributed to the study design, the researched data analysis, the Discussion, manuscript writing and supervision of the manuscript. ARM contributed to ophthalmologic data collection, contributed to the Discussion, made a critical review, and edited the manuscript. JMPH contributed to the study design, s the researched data analysis and the Discussion. ECM contributed to the ophthalmologic data collection, and supervision of the manuscript. All authors read and approved the final manuscript.
